# Characterizing bumble bee (Bombus) communities in the United States and assessing a conservation monitoring method

**DOI:** 10.1002/ece3.4783

**Published:** 2019-01-13

**Authors:** James P. Strange, Amber D. Tripodi

**Affiliations:** ^1^ USDA‐ARS‐Pollinating Insect Biology Management and Systematics Research Unit Logan Utah

**Keywords:** bumble bees, community structure, conservation monitoring, national survey, pollinator diversity, species richness

## Abstract

**Aim:**

Bumble bees (Hymenoptera: Apidae: *Bombus*) are economically and ecologically important pollinators in agroecosystems and wildland habitats. In the Nearctic region, there are approximately 41 species, of which the IUCN lists twelve species as vulnerable, endangered, or critically endangered. We conducted a standardized faunal survey to inform ongoing conservation efforts including petitions under review for the Endangered Species Act. Furthermore, we test the appropriateness of a methodology for accurately sampling bumble bee communities.

**Location:**

The United States of America, including 31 sites in 15 states.

**Methods:**

We surveyed 15 states in the summer of 2015 to assess community composition and relative species abundance at agricultural and seminatural sites throughout the United States. We collected approximately 100 bees, using aerial nets, from each of 31 sites and identified specimens to species, totaling 3,252 bees. We assessed our survey methodology to understand whether it accurately sampled the potential community of bumble bees at each site for utility in future monitoring efforts.

**Results:**

Average site species richness was 5.1 ± 2.05, and we detected 30 of the 41 species documented historically within the contiguous United States. Sampling a site beyond 100 bees rarely added additional species detections, whereas adding additional sampling sites within an ecoregion frequently increased the species richness for the ecoregion. Thirteen of the 30 species we detected each accounted for <1% of the total fauna, and two species accounted for 49.02% of all bees captured. Species richness and evenness increased with increasing latitude across communities.

**Main Conclusions:**

Species diversity and evenness in bumble bees increases in northern latitudes and increasing elevation in the United States; however, a few common species tend to dominate communities while many species occur only in low numbers. The results of this survey effort can inform current conservation evaluations and planning.

## 1 INTRODUCTION

Bumble bees (Hymenoptera: Apidae: *Bombus* Latrielle) are abundant and diverse native social pollinators in the United States. There are approximately 41 bumble bee species that occur in the USA south of Alaska (Williams, Thorp, Richardson, & Colla, 2014). The majority of species are primitively eusocial organisms and form annual colonies that complete a colony reproductive cycle each year; however, several socially parasitic species depend on host species for nesting and reproduction (biology of *Bombus* reviewed in Goulson, 2010). Species have evolved to fill nearly every ecological region in the 48 contiguous states and Alaska; however, they are not native to Hawaii, Puerto Rico, or most of the outlying territories. Additionally, there is a strong signature of higher diversity in cooler and more alpine climates (Williams, 2013).

The distribution of bumble bee species across the landscape of North America is complex, and various geographic and biological constraints tend to define species distributions (Koch, Looney, Sheppard, & Strange, 2017; Lozier, Strange, Stewart, & Cameron, 2011; Williams et al., 2014). In the contiguous 48 states, there exists a strong regional signature in the composition of bumble bee communities. For example, a distinct assemblage of bumble bee species occurs along the Pacific Coast (Koch et al., 2017), and while some of the species also occur east of the Sierra Nevada and Cascade Mountain ranges, six species are mainly restricted to the Pacific coast region (Koch, Strange, & Williams, 2012; Williams et al., 2014). Another group of bumble bees is less geographically restricted, but is more constrained to habitat, occurring only in high mountain, alpine areas in the southwest and reappearing in lower elevations in northern states (Jackson et al., 2018; Lozier, Strange, & Koch, 2013), Canada (Hatten, Strange, & Maxwell, 2015) and Alaska (Koch & Strange, 2012; Williams, 2013).

Among the diverse species in North America, there is considerable variability in species’ vulnerability. Similarly, the various potential drivers of population levels are complex. Worldwide, habitat loss (Goulson, Hanley, Darvill, Ellis, & Knight, 2005), climate change (Kerr et al., 2015; Miller‐Struttmann et al., 2015), pesticides (Goulson, Nicholls, Botias, & Rotheray, 2015), human‐mediated pathogen spread (Arbetman, Meeus, Morales, Aizen, & Smagghe, 2013; Goka, Okabe, Yoneda, & Niwa, 2001; Graystock et al., 2013), and competition with non‐native bees (Morales, Arbetman, Cameron, & Aizen, 2013) have all been cited as potential causes of species decline (Goulson et al., 2005). It is hypothesized that those species with small endemic ranges are at a heightened risk of extinction (Zayed, 2009; Zayed & Packer, 2005), yet in North American *Bombus*, some formerly widespread species seem equally susceptible (Cameron et al., 2011). Despite declines known on both regional and national levels, no unified monitoring protocol for bumble bee species abundance or distribution is currently in place in the United States. While a national approach was taken to survey bumble bees from 2007 to 2010 (Cameron et al., 2011) and developing a national native bee sampling protocol is recognized to be important (Lebuhn et al., 2012, but see Tepedino, Durham, Cameron, & Goodell, 2015 and Lebuhn et al., 2015), most other recent efforts have either been regionally focused (Bushmann & Drummond, 2015; Colla & Packer, 2008; Figueroa & Bergey, 2015; Grixti, Wong, Cameron, & Favret, 2009; Jacobson, Tucker, Mathiasson, & Rehan, 2018; Koch et al., 2017; Tripodi & Szalanski, 2015) or do not include a systematic, contemporary survey component (Jacobson et al., 2018; Kerr et al., 2015).

Currently, the International Union for the Conservation of Nature (IUCN) lists six species of bumble bee in the United States as critically endangered or endangered, and six as vulnerable (IUCN, 2017; Table 1). Additionally, several species are listed as data deficient. Subsequent to this survey, the rusty‐patched bumble bee, *Bombus affinis*, was listed as endangered in the United States under the Endangered Species Act (Federal Register, 2017), but much of the former range of the species remains unsampled. In fact, remnant populations of this species are currently found in suburban and agricultural areas, making these sites high priority for monitoring efforts. In response to the identified gap in data and the need to monitor species that are threatened or endangered, we conducted a survey of bumble bee communities across the United States during the spring and summer of 2015. Here, we present the findings of our systematic survey with notes on the distribution of species, their relative abundance and community‐level analyses. These are intended as a one‐time rapid assessment effort and not as a comprehensive assessment of individual species distributions or seasonal abundance. We perform post hoc analyses of the relationship among sample size, species abundance and diversity and propose a sampling strategy for bumble bees that can capture the diversity and relative abundance of rare bumble bee species in survey and monitoring planning.

**Table 1:** Thirty bumble bee (*Bombus*) species collected in this survey, including the number of sites and ecoregions in which each species was found, the abundance of each species over the entire collection (*N* = 3,252) and IUCN conservation status

Species	Number sites	Number ecoregions	Overall % abundance	IUCN status
*B. appositus*	4	4	1.72	Least Concern
*B. auricomis*	2	2	0.09	Least Concern
*B. bifarius*	2	2	2.31	Least Concern
*B. bimaculatus*	15	9	5.47	Least Concern
*B. borealis*	1	1	0.98	Least Concern
*B. californicus*	4	3	1.51	Vulnerable^a^
*B. caliginosis*	2	1	2.64	Vulnerable
*B. centralis*	3	3	0.25	Least Concern
*B. citrinus*	1	1	0.12	Least Concern
*B. fervidus*	9	7	4.77	Vulnerable^a^
*B. flavidus*	2	2	0.06	Least Concern
*B. flavifrons*	3	3	1.14	Least Concern
*B. griseocollis*	21	13	12.98	Least Concern
*B. huntii*	4	3	2.43	Least Concern
*B. impatiens*	23	14	36.04	Least Concern
*B. insularis*	1	1	0.06	Least Concern
*B. melanopygus*	2	2	0.06	Least Concern
*B. mixtus*	4	4	0.95	Least Concern
*B. morrisoni*	2	2	0.55	Vulnerable
*B. nevadensis*	3	3	0.15	Least Concern
*B. occidentalis*	2	2	0.28	Vulnerable
*B. pensylvanicus*	5	3	4.18	Vulnerable
*B. perplexus*	7	6	1.29	Least Concern
*B. rufocinctus*	6	5	1.78	Least Concern
*B. sandersoni*	3	2	0.58	Least Concern
*B. ternarius*	4	3	4.89	Least Concern
*B. terricola*	4	3	1.29	Vulnerable
*B. vagans*	12	7	4.98	Least Concern
*B. vandykei*	1	1	0.03	Least Concern
*B. vosnesenskii*	4	3	6.40	Least Concern

a *Bombus californicus* and *B. fervidus *are considered con‐specific by the IUCN, but are considered as separate species in this work, following Koch et al. (2012).

## 2 METHODS

**Figure 1:**
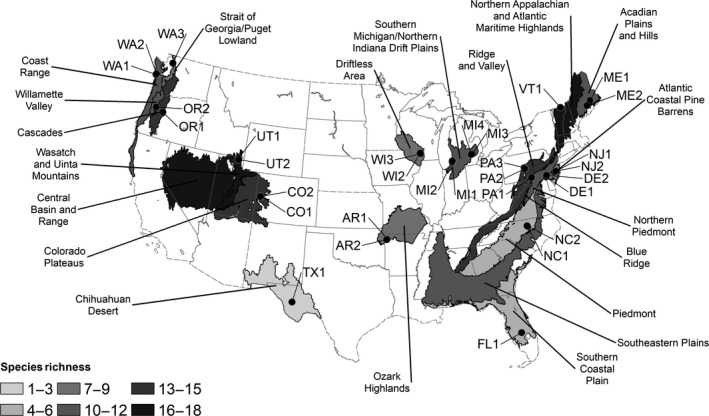
Collection sites (dots) and ecoregions (polygons) sampled during the present survey. Relative species richness within ecoregions is shaded from lowest richness (1–3 species) in light gray to highest richness (16–18 species) in darkest gray. Only surveyed ecoregions are shown

In 2015 (26‐June to 10‐August), we conducted systematic surveys of bumble bees from 31 sites in 15 states (Figure 1). Survey efforts were focused on areas where bumble bees are important for agricultural production and over half of our collections occurred in agricultural landscapes with the majority of other collections being in suburban landscapes adjacent to agricultural areas. Targeted crops included blueberries, tomatoes, strawberries, and cranberries; however, some sites were chosen that had either no history of agricultural production or use of bumble bees for pollination. Nevertheless, these collections generally occurred in regions with agricultural production, with the exception of OR1 and UT2, which occurred in wildlands. Metadata are provided in Supporting Information Appendix S1, and site data are summarized in Supporting Information Appendix S2 with primary and secondary site types listed.

At each site, a collection of approximately 100 foraging bees was taken in a single day between 10:00 and 18:00 local time. Collections occurred in good weather conditions defined as: temperature 15–35°C, no precipitation, <50% cloud cover, and wind speed <15 km/hr. We conducted surveys using two or three collectors using aerial insect nests to capture bumble bees as they foraged on flowering plants for pollen or nectar. Collectors captured foraging bees until a total of 100 worker or male bees were taken at a site, where possible. In most cases, sites were defined as an agricultural field and the field margin directly surrounding the field. However, nonagricultural sites were defined as a patch of flowers not to exceed 5 hectares. Collectors conducted a random walk through the patch or field margins, collecting a bee, stopping to process the bee, then continuing to the next bee they encountered. Netted bees were placed in individual vials and chilled and then given a preliminary field species determination before being killed by freezing on dry ice, except for five sites where time constrains prohibited field identification. Frozen bees were transported back to the USDA‐ARS‐ Pollinating Insect‐ Biology, Management and Systematics Research Unit in Logan, UT where field species identifications were verified or corrected using available taxonomic keys (Koch et al., 2012; Mitchell, 1962; Williams et al., 2014). Specific determinations, sex determination, and site metadata were recorded in a database for further analyses, and species occurrences summarized by site are given in Supporting Information Appendix S3.

All subsequent analyses of community‐level data were performed in R statistical software package version 3.3.2 (R Foundation for Statistical Computing, 2016) unless otherwise noted. We used the VEGAN version 2.5‐2 package (Oksanen et al., 2018) in R to calculate species richness and Shannon's diversity (H), to perform Non‐Metric Dimensional Scaling (NMDS), and to conduct estimates of the species pool. Species pool estimates calculate the total species pool from the sampled species pool for each site, to give an estimate of the number of species that went unsampled (Chao, 1984; Chao, Chazdon, Colwell, & Shen, 2006). All code for calculations is available in Supporting Information Appendix S4 “supplemental scripts.” Pielou's evenness and Jost's Effective Number of Species (ENS) were calculated by hand in spreadsheets (Supporting Information Appendix S2) from values of Shannon's diversity (H) calculated in R. Correlations for species richness with evenness and species richness were tested with a Pearson's correlation in SPSS statistical software.

We further grouped sites by EPA Level III Ecoregion (U.S. Environmental Protection Agency, 2013) and analyzed the diversity within an ecoregion. Data on species occurrences in each of the targeted ecoregions were compiled from eight publications and public databases (Supporting Information Appendix S5) using a synonymized set of species names (Supporting Information Appendix S6) to conform with current taxonomy (Williams et al., 2014). Each species was classified into one of four categories: Present, Occasional, Exotic, and Absent based on occurrence data in the databases (Supporting Information Appendix S7). A “Present” species was defined as a resident species with over 25 specimen records in the assembled data and records <10 years old. An “Occasional” species was defined as a species encountered <25 times in an ecoregion that may occasionally be observed in an ecoregion, but unlikely to be a resident species and not within the last 10 years. An “Exotic” species is one that may have been recorded more than 25 times, but not prior to 10 years ago and these are all records of *B. impatiens *in ecoregions in which it is not native, but has been imported for agricultural pollination. “Absent” species have been recorded fewer than five times in an ecoregion, have no known history of human‐mediated transport, and we consider that any records may be suspect due to disjunction with the remainder of the species range and morphological similarities with species recorded as Present. Further, two categories of Ecoregion Richness were defined. The first, “Liberal Richness,” is the number of Present, Occasional, and Exotic species in an ecoregion, whereas “Conservative Richness” is the number of species designated as Present occurring in an ecoregion.

## 3 RESULTS

A total of 3,252 bees were collected from 31 sites for a mean of 104.9 ± 15.8 *SD* bees per site (Table 1). The most common of the 30 species encountered was *B. impatiens*, the common eastern bumble bee, which comprised 36.04% (*n* = 1,172) of the bees encountered nationwide. Several species were represented by only one (*B. vandykei*) or two (*B. flavidus*, *B. insularis*, and *B. melanopygus*) individuals in the surveys.

The average number of species encountered at sites was 5.01 ± 2.04 *SD* species per site. Two sites (UT2 and VT1) had nine species, whereas two sites had only two species (FL1 and TX1). Correspondingly, VT1 had the highest overall Shannon's diversity (H = 2.00), whereas FL1 had the lowest measure of H (0.054). Species richness was positively correlated with latitude (*r* ^2^ = 0.473, *p* = 0.007, *N* = 31) as was evenness (*r* ^2^ = 0.405, *p* = 0.024, *N* = 31).

**Figure 2:**
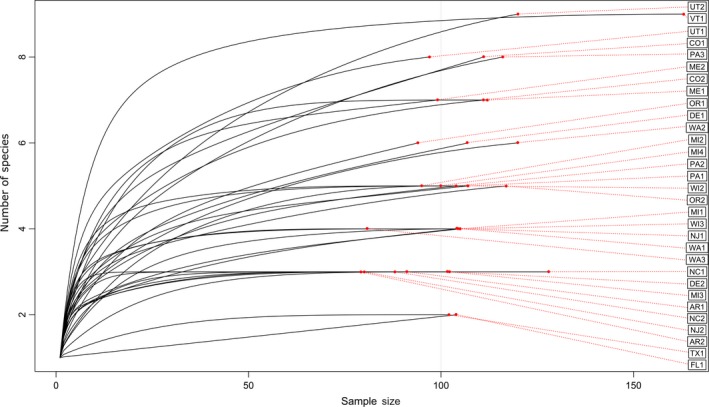
Species accumulation curves generated through rarefaction of sampled data. Endpoints of black lines indicate the number of bees sampled at each site with site labels on the far right and the number of species detected on the *y*‐axis.

Rarefaction of number of species found and the number of bees sampled per site resulted in 31 species accumulation curves (Figure 2) to estimate community richness (Gotelli & Colwell, 2001). Additionally, site samples are displayed with the number of species sampled, the Chao estimator of the species pool (Chao, 1984), and the conservative richness (the number of species historically detected as Present) in the ecoregion (Table 2). The NMDS utilizing species by site (Supporting Information Appendix S8a) identified four major regional clusters of bumble bee communities: northeast, eastern, Rocky Mountains, and west coast Supporting Information Appendix S8a, with several communities falling outside of these broad regional clusters (one site in the Cascade Mountains and one in Texas). When the data are analyzed by ecoregion (Supporting Information Appendix S8b), the clusters largely remain the same; however, the Texas site is then included in the eastern cluster.

**Table 2:** Sites surveyed by state, detected species richness, an estimation of the species pool as calculated following Chao (1984) and an estimation of the number of bumble bee species that remained unsampled at each site. Ecoregion names and ecoregion richness are provided

Site	Richness	Species pool	Unsampled species	Ecoregion	EcoReg richness
AR1	3	3	0	Ozark Highlands	7
AR2	3	3	0	Ozark Highlands	7
CO1	8	11	3	Colorado Plateaus	14
CO2	7	7	0	Colorado Plateaus	14
DE1	6	7	1	Southeastern Plains	10
DE2	3	3	0	Northern Piedmont	10
FL1	2	2	0	Southern Coastal Plain	6
ME1	7	7	0	Acadian Plains and Hills	10
ME2	7	7	0	Acadian Plains and Hills	10
MI1	4	4	0	S. Michigan/N. Indiana Drift Plains	12
MI2	5	5	0	S. Michigan/N. Indiana Drift Plains	12
MI3	3	3	0	S. Michigan/N. Indiana Drift Plains	12
MI4	5	5	0	S. Michigan/N. Indiana Drift Plains	12
NC1	3	3	0	Piedmont	6
NC2	3	3	0	Piedmont	6
NJ1	4	4	0	Atlantic Coastal Pine Barrens	8
NJ2	3	3	0	Atlantic Coastal Pine Barrens	8
OR1	6	7	1	Cascades	13
OR2	5	5	0	Willamette Valley	11
PA1	5	5	0	Blue Ridge	11
PA2	5	5	0	Ridge and Valley	15
PA3	8	8.33	0.33	Ridge and Valley	15
TX1	2	2	0	Chihuahuan Desert	1
UT1	8	8	0	Central Basin and Range	16
UT2	9	9.25	0.25	Wasatch and Uinta Mountains	18
VT1	9	9	0	N. App. & Atl. Maritime Highlands	16
WA1	4	4	0	Coast Range	12
WA2	6	6	0	Coast Range	12
WA3	4	4	0	Strait of Georgia/Puget Lowland	7
WI2	5	5	0	Driftless Area	9
WI3	4	4	0	Driftless Area	9

## 4 DISCUSSION

Bumble bee communities varied widely and reflected the diverse landscapes and land uses of the areas we surveyed. Because our efforts were focused on areas in or near agricultural production, some bumble bee communities are not represented in this work. For example, high‐elevation communities throughout the USA were not sampled. We sampled in several regions that are underrepresented in the recent literature, but there remain gaps in recent sampling effort. Additionally, the sampling effort was directed at mid‐season to maximize the diversity of species encountered, thus early‐season specialists (e.g., *B. bimaculatus*; *B. melanopygus*; Williams et al., 2014) may be underrepresented relative to later emerging species.

Our 31 sites occupied 20 Level III Ecoregions, and the ecoregion proved to be an informative way to view bumble bee communities. Sites in Washington and Oregon (WA1–3 and OR2) captured the unique assemblage of species along the Pacific Coast that has previously been noted (Koch et al., 2017), with sites in the Coast Range, Willamette Valley, and Strait of Georgia/Puget Lowland ecoregions uniquely sharing *B. vosnesenskii *as the most common species (Supporting Information Appendix S8). However, *B. caliginosus *was the second most common in both of the Coast Range sites (WA1 and WA2), yet this species was entirely absent from sites in other ecoregions of the Pacific Northwest. Throughout this work, we find that community distinctions based on ecoregion are much more informative than geographic distance. In the Pacific Northwest, for example, the community within the OR2 site is much more similar to the Washington sites (WA1–3) than the much closer OR1 site that lies within in the Cascades ecoregion (Figure 1, Supporting Information Appendix S8). In the Cascades, *B. vosnesenskii *is absent, and *B. bifarius*, which was absent from the Coast Range, Willamette Valley and Strait of Georgia/Puget Lowland ecoregions, becomes the most common species sampled (Table 2). Similarly, the Utah community sampled in the Wasatch and Uinta Mountains (UT2) is quite distinct from the community sampled in nearby Central Basin and Range (UT1), which shares a community most similar to those in the Colorado Plateaus (CO1, CO2; Figure 1, Supporting Information Appendix S8). Recognizing the similarities and differences among bumble bee communities in different ecoregions can help guide sampling strategies.

The current assessment of bumble bee communities at 31 sites in 15 states can be used to understand both current distribution and status of several species, including the three species under USFWS Endangered Species Act review (Federal Register, 2016) and the recently listed *B. affinis *(Federal egister, 2017). For example, we surveyed at 16 sites in the historic range of the endangered *B. affinis* and did not find any specimens of that species. Thus, the widespread decline of the species appears to be persistent. Conversely, another species that is listed as vulnerable by the IUCN (IUCN, 2017), *B. fervidus*, was found at nearly a third of all sites (N sites = 9) and was the most abundant bee at two sites (CO1 and UT1) and the third most abundant bee at three other sites (CO2, DE1, and PA1). Similarly, *B. californicus* (considered to be conspecific with *B. fervidus *in the IUCN Red List) was found in three ecoregions at four sites and was the second most abundant species at OR2 and the third most common at WA1. Nationwide, if we consider the species as IUCN does, *fervidus*/*californicus* is the fourth most abundant species we encountered. The broad distribution of the species and the high relative abundance suggest that further investigation of the species status is warranted. It may be that the vulnerable status given to this group was driven more by anecdotal evidence than by actual survey data, that a recent short‐term trend in *B. fervidus* populations was driving this assessment, or that areas in which these species are common were inconsistently sampled over time. Finally, it may be that our data represent a particularly “good” year for this species complex, and that in fact, the vulnerable assessment is accurate. We expect that the current data and resampling will inform assessment and policy making for these species going forward.

Also of note is that data similar to ours may be useful in determining areas where possible species invasions have a potential to occur. For example, in both west Texas and western Colorado, we found *B. impatiens* foraging outside of local greenhouses. This species, sold commercially for greenhouse‐grown tomato pollination (Velthuis & van Doorn, 2006), is not native this far west (Williams et al., 2014), but is now freely foraging among the native western bees at some sites; although we did not detect the species in the Pacific Northwest where it had previously been seen outside of containment (Ratti & Colla, 2010). While foraging outside of a greenhouse does not mean the species is locally established, this does suggest the potential for establishment of this species in novel areas. Our data from west Texas (TX1) and western Colorado (CO1), where we surveyed within a kilometer of a commercial greenhouses in both states, suggest that *B. impatiens *can be found at the point of use, but was not detected in the landscape ten kilometers away (CO2). We suggest that systematic surveys in areas with large greenhouse industries (e.g., British Columbia, Washington, and southern California) should take place to monitor for potential invasions by this non‐native species (Aizen, et al., 2018). Evidence of establishment of this non‐native species in British Columbia (Ratti & Colla, 2010) and our records around greenhouses (CO1 and TX1) indicate that diligence is warranted.

Despite new data, clearly there are geographic areas that were not well sampled and time periods throughout the season that lack sufficient investigation, thus precaution should be taken in interpreting these results too broadly. For example, species of bumble bees have different seasonal abundances, and studies sampling an area during a single visit, like ours, are likely to underestimate the true, local diversity. In some cases, we encountered mostly males from one species (indicating colonies were at a mature phase) and workers of another species (indicating colonies were still growing and had not yet begun reproducing). Sampling earlier or later in the season would likely result in different assessments of species abundance. In addition to sampling more geographic areas, we recommend that bumble bee conservationists target survey locations multiple times throughout the active period of the genus before drawing conclusions about the community composition.

Evaluating our sampling scheme through rarefaction of species accumulation and species pool estimates provides an assessment of this as a reasonable sampling technique for bumble bee community surveys. We find that over half our sites reach an asymptote in the rarefaction curves by the time 100 bumble bees are sampled, with many asymptotic by a sample size of 75 or less (Figure 2). Further, at all but five of the sites sampled, we estimated that the full species pool of bumble bees was detected (Table 2, Chao, 1984). It should be noted that while these results indicate that the sampling scheme is sufficient to capture most species at most sites (Gotelli & Colwell, 2001), single site sampling within an ecoregion generally falls short of detecting all the species that occur within that ecoregion based on published data (Table 2, Supporting Information Appendix S5). Even in the nine ecoregions where we sampled multiple sites, we only detected all the possible species in one ecoregion with low diversity (Chihuahuan Desert). However, sampling two or more sites within an ecoregion increased the number of species we detected by at least one species in five of nine cases (Table 2), thus focusing effort on multiple habitat types within an ecoregion could expand the sampled species pool.

Sampling schemes that are aimed at detecting particularly rare species (e.g., *B. affinis, B. occidentalis*) will need to be tailored to achieve the precision desired for the goals of the project. The problem of rare species detection could, again, be mitigated by multiple sampling dates in a year or by increasing sample size where rare species are suspected, and the sampling scheme should be reevaluated regularly to insure that precision is maintained. Alternatively, a sequential sampling scheme with stopping rules could be employed (Chao & Jost, 2012); however, that would require that accurate field identification and the subsequent analysis of the power of the sampling scheme take place in the field. As our data indicate, our success at field identifications was variable across communities, with an average identification error rate of 7.9%. The usefulness of any sampling scheme will have to balance minimizing effort with the need for precision.

As efforts to survey and monitor bumble bees gain prominence in the wake of the Endangered Species Act listing of *B. affinis*, it will be critical to standardize the sampling protocols to determine population levels and community structure. Similarly, both historic data and contemporary surveys are required to compare the changes in bumble bee community composition, and thus capture both short‐ and long‐term population trends. A standardized and serious effort to document the range of species, the current status and important site characteristics (biotic and abiotic) that affect species occurrence will be necessary to determine the effectiveness of conservation efforts. Our current study both adds data to data‐deficient areas and underscores that other regions remain difficult to survey and need specific attention.

## CONFLICT OF INTEREST

The authors declare no conflicts of interest in this work. USDA is an equal opportunity provider and employer.

## AUTHOR CONTRIBUTIONS

JPS and ADT conceived the research, led field sampling, identified specimens, and analyzed the data. JPS was primarily responsible for writing with significant input from ADT. ADT wrote the code for analyses in R.

## Data Availability

All specimen data used in this study are available to the public through the U.S. National Pollinating Insects Collection (NPIC) database and the BISON database https://bison.usgs.gov/#home.
