# Supplementary material for: Characterizing bumble bee (Bombus) communities in the United States and assessing a conservation monitoring method
Source: Ecol Evol. 2019 Jan 13;9(3):1061–9. doi: 10.1002/ece3.4783 (PMC6374645; doi:10.1002/ece3.4783)
Supplement: Supplementary file 1 [file ECE3-9-1061-s001.docx]

| **Title:** | **Characterizing bumble bee (*Bombus*) communities in the United States and assessing a conservation monitoring method** |
| --- | --- |
| **Authors:** | J. P. Strange and A. D. Tripodi |
| **Journal:** | Ecology and Evolution |
| **Contact Info:** | [james.strange@ars.usda.gov](mailto:james.strange@ars.usda.gov) |
| **Data accessibility:** | All specimen data used in this study are available to the public through the U.S. National Pollinating Insects Collection (NPIC) database and the USGS BISON database <https://bison.usgs.gov/#home>. |
| **Worksheet Name** | **Description** |
| **Appendix2_SiteData** | Details about the collection sites, by site code, Latitude, Longitude, total collected at each site, and the Level III Ecoregion containing each site. |
| **Appendix3_SppSite** | Counts of individuals of each species (truncated to first six letters) collected at each site (given by site code), including totals and species richness. Effective Number of Species (ENS), Shannon’s diversity (H), Pielou’s Evenness, and the field identification error rates are given for each site. |
| **Appendix5_DataSources** | Sources of records used to determine SppOccur. |
| **Appendix6_Synonyms** | Taxonomic designations applied to records. |
| **Appendix7_SppOccur** | The occurrence of each species in each Level III Ecoregion included in this work. Present = a resident species with over 25 specimen records in the assembled data and records less than 10 years old, Occasional = a species encountered < 25 times in an ecoregion that may occasionally be observed in an ecoregion, but unlikely to be a resident species, Exotic = records of *Bombus impatiens* in an ecoregion in which it is not native, but has been imported for agricultural pollination. Liberal Richness = the number of Present, Occasional, and Exotic species in an ecoregion; Conservative Richness = the number of species designated as Present occurring in an ecoregion. |
| **Appendix8_NMDS** | Output from NMDS analysis in two graphics. Appendix 8a is an NMDS comparing the species composition of sites by the species detected at those sites. Appendix 8b is an NMDS of the data grouped by ecoregion to compare the species composition detected at that level. |

Appendix1_Metadata
