# Supplementary material for: Characterizing bumble bee (Bombus) communities in the United States and assessing a conservation monitoring method
Source: Ecol Evol. 2019 Jan 13;9(3):1061–9. doi: 10.1002/ece3.4783 (PMC6374645; doi:10.1002/ece3.4783)
Supplement: Supplementary file 2 [file ECE3-9-1061-s002.docx]

| **SiteCode** | **Lat** | **Long** | **N** | **Level3EcoRegion** | **SiteType** | **SecondayType** |
| --- | --- | --- | --- | --- | --- | --- |
| AR1 | 36.101000 | -94.172000 | 91 | Ozark Highlands | Mixed Agricultural | |
| AR2 | 36.067094 | -94.233578 | 78 | Ozark Highlands | Prairie | Mixed Agricultural |
| CO1 | 39.083170 | -108.427000 | 111 | Colorado Plateaus | Mixed Agricultural | |
| CO2 | 39.035460 | -108.540000 | 111 | Colorado Plateaus | Suburban Garden | Mixed Agricultural |
| DE1 | 39.667800 | -75.747200 | 107 | Southeastern Plains | Mixed Agricultural | Suburban |
| DE2 | 39.787570 | -75.649200 | 102 | Northern Piedmont | Suburban Garden | Hardwood Forest |
| FL1 | 27.318200 | -81.367300 | 104 | Southern Coastal Plain | Suburban Garden | Mixed Agricultural |
| ME1 | 44.858630 | -68.316400 | 112 | Acadian Plains and Hills | Mixed Agricultural | |
| ME2 | 44.526000 | -68.869000 | 99 | Acadian Plains and Hills | Suburban Garden | Mixed Agricultural |
| MI1 | 42.179430 | -86.258700 | 105 | Southern Michigan/Northern Indiana Drift Plains | Suburban Garden | Mixed Agricultural |
| MI2 | 42.263210 | -86.229500 | 95 | Southern Michigan/Northern Indiana Drift Plains | Mixed Agricultural | |
| MI3 | 42.630630 | -83.747600 | 101 | Southern Michigan/Northern Indiana Drift Plains | Mixed Agricultural | |
| MI4 | 42.606532 | -83.903508 | 100 | Southern Michigan/Northern Indiana Drift Plains | Mixed Agricultural | |
| NC1 | 35.926680 | -78.855300 | 128 | Piedmont | Suburban Garden | Mixed Agricultural |
| NC2 | 36.001880 | -78.932200 | 88 | Piedmont | Suburban Garden | Mixed Agricultural |
| NJ1 | 39.911010 | -74.701100 | 104 | Atlantic Coastal Pine Barrens | Mixed Agricultural | |
| NJ2 | 39.953090 | -74.500000 | 80 | Atlantic Coastal Pine Barrens | Mixed Agricultural | |
| OR1 | 44.224330 | -121.872000 | 94 | Cascades | Wet Meadow | Evergreen Forest |
| OR2 | 44.474000 | -122.839000 | 117 | Willamette Valley | Mixed Agricultural | Suburban |
| PA1 | 39.867250 | -77.376200 | 107 | Blue Ridge | Mixed Agricultural | |
| PA2 | 40.710540 | -77.945000 | 104 | Ridge and Valley | Mixed Agricultural | |
| PA3 | 40.798310 | -78.002500 | 116 | Ridge and Valley | Suburban | Mixed Agricultural |
| TX1 | 30.598230 | -103.909000 | 102 | Chihuahuan Desert | Mixed Agricultural | |
| UT1 | 41.756820 | -111.807000 | 97 | Central Basin and Range | Mixed Agricultural | Suburban |
| UT2 | 41.797200 | -111.647000 | 119 | Wasatch and Uinta Mountains | Mountain Meadow | Evergreen Forest |
| VT1 | 44.986770 | -72.075200 | 163 | Northern Appalachian and Atlantic Maritime Highlands | Mixed Agricultural | |
| WA1 | 47.042600 | -124.065000 | 103 | Coast Range | Mixed Agricultural | Evergreen Forest |
| WA2 | 47.121290 | -124.038000 | 120 | Coast Range | Mixed Agricultural | Evergreen Forest |
| WA3 | 48.372020 | -122.401000 | 81 | Strait of Georgia/Puget Lowland | Mixed Agricultural | Evergreen Forest |
| WI2 | 43.150890 | -89.990300 | 107 | Driftless Area | Mixed Agricultural | Suburban |
| WI3 | 43.078250 | -89.905600 | 105 | Driftless Area | Mixed Agricultural | Suburban |

Appendix2_SiteData. Collection sites, by Site Code, Latitude, Longitude, total bees collected at each site (N), and the US-EPA Level III Ecoregion containing each site. Site Type and Secondary Type were assessed by collectors in the field and are not quantitatively defined.
