# Supplementary material for: Characterizing bumble bee (Bombus) communities in the United States and assessing a conservation monitoring method
Source: Ecol Evol. 2019 Jan 13;9(3):1061–9. doi: 10.1002/ece3.4783 (PMC6374645; doi:10.1002/ece3.4783)
Supplement: Supplementary file 3 [file ECE3-9-1061-s003.docx]

Appendix3_SppSite. Counts of individuals of each species (truncated to first six letters) collected at each site (given by site code), including totals and species richness. Effective Number of Species (ENS), Shannon’s diversity (H), Pielou’s Evenness, and the field identification error rates are given for each site.

| **Species** | **AR1** | **AR2** | **CO1** | **CO2** | **DE1** | **DE2** | **FL1** | **ME1** | **ME2** | **MI1** | **MI2** | **MI3** | **MI4** | **NC1** | **NC2** | **NJ1** | **NJ2** | **OR1** | **OR2** | **PA1** | **PA2** | **PA3** | **TX1** | **UT1** | **UT2** | **VT1** | **WA1** | **WA2** | **WA3** | **WI2** | **WI3** | **Totals** |
| --- | --- | --- | --- | --- | --- | --- | --- | --- | --- | --- | --- | --- | --- | --- | --- | --- | --- | --- | --- | --- | --- | --- | --- | --- | --- | --- | --- | --- | --- | --- | --- | --- |
| *appositus* | 0 | 0 | 1 | 0 | 0 | 0 | 0 | 0 | 0 | 0 | 0 | 0 | 0 | 0 | 0 | 0 | 0 | 0 | 2 | 0 | 0 | 0 | 0 | 3 | 50 | 0 | 0 | 0 | 0 | 0 | 0 | **56** |
| *auricomis* | 0 | 0 | 0 | 0 | 1 | 0 | 0 | 0 | 0 | 0 | 0 | 0 | 0 | 0 | 0 | 0 | 0 | 0 | 0 | 0 | 0 | 2 | 0 | 0 | 0 | 0 | 0 | 0 | 0 | 0 | 0 | **3** |
| *bifarius* | 0 | 0 | 0 | 0 | 0 | 0 | 0 | 0 | 0 | 0 | 0 | 0 | 0 | 0 | 0 | 0 | 0 | 73 | 0 | 0 | 0 | 0 | 0 | 0 | 2 | 0 | 0 | 0 | 0 | 0 | 0 | **75** |
| *bimaculatus* | 0 | 0 | 0 | 0 | 3 | 0 | 0 | 7 | 14 | 4 | 13 | 0 | 4 | 33 | 4 | 1 | 0 | 0 | 0 | 7 | 60 | 12 | 0 | 0 | 0 | 8 | 0 | 0 | 0 | 6 | 2 | **178** |
| *borealis* | 0 | 0 | 0 | 0 | 0 | 0 | 0 | 0 | 0 | 0 | 0 | 0 | 0 | 0 | 0 | 0 | 0 | 0 | 0 | 0 | 0 | 0 | 0 | 0 | 0 | 32 | 0 | 0 | 0 | 0 | 0 | **32** |
| *californicus* | 0 | 0 | 0 | 0 | 0 | 0 | 0 | 0 | 0 | 0 | 0 | 0 | 0 | 0 | 0 | 0 | 0 | 0 | 27 | 0 | 0 | 0 | 0 | 0 | 0 | 0 | 17 | 2 | 3 | 0 | 0 | **49** |
| *caliginosis* | 0 | 0 | 0 | 0 | 0 | 0 | 0 | 0 | 0 | 0 | 0 | 0 | 0 | 0 | 0 | 0 | 0 | 0 | 0 | 0 | 0 | 0 | 0 | 0 | 0 | 0 | 32 | 54 | 0 | 0 | 0 | **86** |
| *centralis* | 0 | 0 | 0 | 1 | 0 | 0 | 0 | 0 | 0 | 0 | 0 | 0 | 0 | 0 | 0 | 0 | 0 | 0 | 0 | 0 | 0 | 0 | 0 | 2 | 5 | 0 | 0 | 0 | 0 | 0 | 0 | **8** |
| *citrinus* | 0 | 0 | 0 | 0 | 0 | 4 | 0 | 0 | 0 | 0 | 0 | 0 | 0 | 0 | 0 | 0 | 0 | 0 | 0 | 0 | 0 | 0 | 0 | 0 | 0 | 0 | 0 | 0 | 0 | 0 | 0 | **4** |
| *fervidus* | 0 | 0 | 60 | 24 | 12 | 0 | 0 | 0 | 0 | 1 | 0 | 0 | 2 | 0 | 0 | 3 | 0 | 0 | 0 | 19 | 0 | 0 | 0 | 30 | 0 | 0 | 4 | 0 | 0 | 0 | 0 | **155** |
| *flavidus* | 0 | 0 | 0 | 0 | 0 | 0 | 0 | 0 | 0 | 0 | 0 | 0 | 0 | 0 | 0 | 0 | 0 | 1 | 0 | 0 | 0 | 0 | 0 | 0 | 1 | 0 | 0 | 0 | 0 | 0 | 0 | **2** |
| *flavifrons* | 0 | 0 | 0 | 0 | 0 | 0 | 0 | 0 | 0 | 0 | 0 | 0 | 0 | 0 | 0 | 0 | 0 | 3 | 0 | 0 | 0 | 0 | 0 | 0 | 0 | 0 | 0 | 2 | 32 | 0 | 0 | **37** |
| *griseocollis* | 15 | 51 | 4 | 43 | 16 | 42 | 1 | 0 | 0 | 39 | 29 | 6 | 8 | 36 | 25 | 31 | 11 | 0 | 14 | 3 | 0 | 13 | 0 | 22 | 0 | 0 | 0 | 0 | 0 | 6 | 7 | **422** |
| *huntii* | 0 | 0 | 17 | 34 | 0 | 0 | 0 | 0 | 0 | 0 | 0 | 0 | 0 | 0 | 0 | 0 | 0 | 0 | 0 | 0 | 0 | 0 | 0 | 22 | 6 | 0 | 0 | 0 | 0 | 0 | 0 | **79** |
| *impatiens* | 61 | 24 | 11 | 0 | 74 | 56 | 103 | 7 | 11 | 61 | 35 | 92 | 84 | 59 | 59 | 69 | 64 | 0 | 0 | 35 | 35 | 82 | 4 | 0 | 0 | 6 | 0 | 0 | 0 | 51 | 89 | **1172** |
| *insularis* | 0 | 0 | 0 | 0 | 0 | 0 | 0 | 0 | 0 | 0 | 0 | 0 | 0 | 0 | 0 | 0 | 0 | 0 | 0 | 0 | 0 | 0 | 0 | 0 | 2 | 0 | 0 | 0 | 0 | 0 | 0 | **2** |
| *melanopygus* | 0 | 0 | 1 | 0 | 0 | 0 | 0 | 0 | 0 | 0 | 0 | 0 | 0 | 0 | 0 | 0 | 0 | 1 | 0 | 0 | 0 | 0 | 0 | 0 | 0 | 0 | 0 | 0 | 0 | 0 | 0 | **2** |
| *mixtus* | 0 | 0 | 0 | 0 | 0 | 0 | 0 | 0 | 0 | 0 | 0 | 0 | 0 | 0 | 0 | 0 | 0 | 8 | 1 | 0 | 0 | 0 | 0 | 0 | 0 | 0 | 0 | 6 | 16 | 0 | 0 | **31** |
| *morrisoni* | 0 | 0 | 0 | 3 | 0 | 0 | 0 | 0 | 0 | 0 | 0 | 0 | 0 | 0 | 0 | 0 | 0 | 0 | 0 | 0 | 0 | 0 | 0 | 15 | 0 | 0 | 0 | 0 | 0 | 0 | 0 | **18** |
| *nevadensis* | 0 | 0 | 1 | 0 | 0 | 0 | 0 | 0 | 0 | 0 | 0 | 0 | 0 | 0 | 0 | 0 | 0 | 0 | 0 | 0 | 0 | 0 | 0 | 2 | 2 | 0 | 0 | 0 | 0 | 0 | 0 | **5** |
| *occidentalis* | 0 | 0 | 0 | 0 | 0 | 0 | 0 | 0 | 0 | 0 | 0 | 0 | 0 | 0 | 0 | 0 | 0 | 8 | 0 | 0 | 0 | 0 | 0 | 0 | 1 | 0 | 0 | 0 | 0 | 0 | 0 | **9** |
| *pensylvanicus* | 15 | 3 | 16 | 4 | 0 | 0 | 0 | 0 | 0 | 0 | 0 | 0 | 0 | 0 | 0 | 0 | 0 | 0 | 0 | 0 | 0 | 0 | 98 | 0 | 0 | 0 | 0 | 0 | 0 | 0 | 0 | **136** |
| *perplexus* | 0 | 0 | 0 | 0 | 1 | 0 | 0 | 4 | 5 | 0 | 3 | 0 | 0 | 0 | 0 | 0 | 0 | 0 | 0 | 0 | 3 | 0 | 0 | 0 | 0 | 25 | 0 | 0 | 0 | 1 | 0 | **42** |
| *rufocinctus* | 0 | 0 | 0 | 2 | 0 | 0 | 0 | 0 | 0 | 0 | 0 | 0 | 0 | 0 | 0 | 0 | 0 | 0 | 0 | 0 | 1 | 1 | 0 | 1 | 51 | 2 | 0 | 0 | 0 | 0 | 0 | **58** |
| *sandersoni* | 0 | 0 | 0 | 0 | 0 | 0 | 0 | 4 | 1 | 0 | 0 | 0 | 0 | 0 | 0 | 0 | 0 | 0 | 0 | 0 | 0 | 0 | 0 | 0 | 0 | 14 | 0 | 0 | 0 | 0 | 0 | **19** |
| *ternarius* | 0 | 0 | 0 | 0 | 0 | 0 | 0 | 73 | 56 | 0 | 0 | 0 | 0 | 0 | 0 | 0 | 0 | 0 | 0 | 0 | 0 | 3 | 0 | 0 | 0 | 27 | 0 | 0 | 0 | 0 | 0 | **159** |
| *terricola* | 0 | 0 | 0 | 0 | 0 | 0 | 0 | 13 | 7 | 0 | 0 | 0 | 0 | 0 | 0 | 0 | 0 | 0 | 0 | 0 | 0 | 1 | 0 | 0 | 0 | 21 | 0 | 0 | 0 | 0 | 0 | **42** |
| *vagans* | 0 | 0 | 0 | 0 | 0 | 0 | 0 | 4 | 5 | 0 | 15 | 3 | 2 | 0 | 0 | 0 | 5 | 0 | 0 | 43 | 5 | 2 | 0 | 0 | 0 | 28 | 0 | 0 | 0 | 43 | 7 | **162** |
| *vandykei* | 0 | 0 | 0 | 0 | 0 | 0 | 0 | 0 | 0 | 0 | 0 | 0 | 0 | 0 | 0 | 0 | 0 | 0 | 0 | 0 | 0 | 0 | 0 | 0 | 0 | 0 | 0 | 1 | 0 | 0 | 0 | **1** |
| *vosnesenskii* | 0 | 0 | 0 | 0 | 0 | 0 | 0 | 0 | 0 | 0 | 0 | 0 | 0 | 0 | 0 | 0 | 0 | 0 | 73 | 0 | 0 | 0 | 0 | 0 | 0 | 0 | 50 | 55 | 30 | 0 | 0 | **208** |
| **Total by site** | **91** | **78** | **111** | **111** | **107** | **102** | **104** | **112** | **99** | **105** | **95** | **101** | **100** | **128** | **88** | **104** | **80** | **94** | **117** | **107** | **104** | **116** | **102** | **97** | **120** | **163** | **103** | **120** | **81** | **107** | **105** | **3252** |
| **Richness** | **3** | **3** | **8** | **7** | **6** | **3** | **2** | **7** | **7** | **4** | **5** | **3** | **5** | **3** | **3** | **4** | **3** | **6** | **5** | **5** | **5** | **8** | **2** | **8** | **9** | **9** | **4** | **6** | **4** | **5** | **4** |  |
| **ENS** | **2.37** | **2.15** | **3.96** | **1.91** | **2.64** | **2.27** | **1.06** | **3.43** | **3.97** | **2.34** | **4.07** | **1.43** | **1.88** | **2.90** | **2.15** | **2.18** | **1.87** | **2.28** | **2.71** | **3.73** | **2.66** | **2.83** | **1.18** | **5.15** | **3.65** | **7.43** | **3.12** | **2.84** | **3.25** | **2.96** | **1.78** |  |
| **H Shannon's Diversity Index** | **0.86** | **0.77** | **1.38** | **0.65** | **0.97** | **0.82** | **0.05** | **1.23** | **1.38** | **0.85** | **1.40** | **0.36** | **0.63** | **1.06** | **0.77** | **0.78** | **0.62** | **0.82** | **1.00** | **1.32** | **0.98** | **1.04** | **0.17** | **1.64** | **1.30** | **2.01** | **1.14** | **1.04** | **1.18** | **1.09** | **0.58** |  |
| **Pielou’s Evenness** | **0.79** | **0.70** | **0.66** | **0.33** | **0.54** | **0.75** | **0.08** | **0.63** | **0.71** | **0.61** | **0.87** | **0.33** | **0.39** | **0.97** | **0.70** | **0.56** | **0.57** | **0.46** | **0.62** | **0.82** | **0.61** | **0.50** | **0.24** | **0.79** | **0.59** | **0.91** | **0.82** | **0.58** | **0.85** | **0.68** | **0.42** |  |
| **Field ID Error (%)** | **ND** | **ND** | **ND** | **ND** | **7.5** | **6.9** | **1** | **11.6** | **6.1** | **6.7** | **6.3** | **0** | **0** | **2.3** | **4.5** | **4.8** | **7.5** | **3.2** | **0** | **0** | **15.4** | **ND** | **0** | **18.6** | **10.8** | **25.2** | **13.6** | **25** | **6.2** | **8.4** | **1.9** | **7.9** |
