# Supplementary material for: Characterizing bumble bee (Bombus) communities in the United States and assessing a conservation monitoring method
Source: Ecol Evol. 2019 Jan 13;9(3):1061–9. doi: 10.1002/ece3.4783 (PMC6374645; doi:10.1002/ece3.4783)
Supplement: Supplementary file 5 [file ECE3-9-1061-s005.docx]

| **Sources of data used to construct the SppOccur table** |
| --- |
| Gibbs, J, JS Ascher, MG Rightmyer, & R Isaacs. 2017. The bees of Michigan (Hymenoptera: Apoidea: Anthophila), with notes on distribution, taxonomy, pollination, and natural history. Zootaxa, 4352(1), 1-160. doi: 10.11646/zootaxa.4352.1.1. |
| Beckham, JL & S Atkinson. 2017. An updated understanding of Texas bumble bee (Hymenoptera: Apidae) species presence and potential distributions in Texas, USA. PeerJ, 5: e3612. doi: 10.7717/peerj.3612. |
| Biodiversity Information Serving Our Nation (BISON), Available at https://bison.usgs.gov, Accessed 31-Dec-2017. |
| Discover Life in America Great Smoky Mountains National Park All Taxa Biodiversity Inventory, Available at 05-Dec-2017https://www.dlia.org/atbidata/SpeciesList.php?taxon=OrderName&tname=Hymenoptera&wshed=All%20Watersheds, Accessed 05-Dec-2017. |
| Koch, JB, J Lozier, JP Strange, H Ikerd, T Griswold, N Cordes, L Solter, I Stewart, & SA Cameron. 2015. USBombus, a database of contemporary survey data for North American Bumble Bees (Hymenoptera, Apidae, *Bombus*) distributed in the United States. Biodiversity data journal 3: e6833. doi: 10.3897/BDJ.3.e6833. |
| Symbiota Collections of Arthropods Network (SCAN), Available at http://scan-bugs.org/portal/index.php, Accessed on 23-Dec-2017. |
| Thorp, RW & DS Horning. 1983. Bumble bees and cuckoo bumble bees of California (Hymenoptera, Apidae). Bulletin of the California Insect Survey, Vol. 23. Univ of California Press, 79 pgs. |
| Tripodi, AD & AL Szalanski. 2015. The bumble bees (Hymenoptera: Apidae: *Bombus*) of Arkansas, fifty years later. *Journal of Melittology*, 50: 42752. doi: 10.17161/jom.v0i50.4834. |

Appendix5_DataSources.
