# Supplementary material for: Characterizing bumble bee (Bombus) communities in the United States and assessing a conservation monitoring method
Source: Ecol Evol. 2019 Jan 13;9(3):1061–9. doi: 10.1002/ece3.4783 (PMC6374645; doi:10.1002/ece3.4783)
Supplement: Supplementary file 6 [file ECE3-9-1061-s006.docx]

Appendix6_Synonyms. List of taxonomic names used in DataSources synonymized to reflect current taxonomy as used in the present study.

| **Reported** | **ReplacedWith** |
| --- | --- |
| *alaskensis* | *flavifrons* |
| *alboanalis* | *jonellus* |
| *americanorum* | *pensylvanicus* |
| *appostius* | *appositus* |
| *ashtoni* | *bohemicus* |
| *caliginosa* | *caliginosus* |
| *consimilis* | *vagans* |
| *dimidiatus* | *flavifrons* |
| *dubius* | *californicus* |
| *edwardsii* | *melanopygus* |
| *edwardsii* var. *kenoyeri* | *bifarius* |
| *fernaldae* | *flavidus* |
| *fervida* | *fervidus* |
| *fervidus* | *fervidus* |
| *fervidus dorsalis* | *fervidus* |
| *gelidus* | *sylvicola* |
| *howardi* | *occidentalis* |
| *improbus* | *nevadensis* |
| *intrudens* | *variabilis* |
| *iridis* | *rufocinctus* |
| *iridis* var. *phaceliae* | *rufocinctus* |
| *juxtus* | *centralis* |
| *kincaidii* | *polaris* |
| *mckayi* | *occidentalis* |
| *mixtuosus* | *sitkensis* |
| *moderatus* | *cryptarum* |
| *monardae* | *centralis* |
| *mormonorum* | *griseocollis* |
| *nevadensis auricomus* | *auricomus* |
| *nevandensis* | *nevadensis* |
| *nigrocinctus* | *crotchii* |
| *occidentalis nigroscutatus* | *occidentalis* |
| *oregonensis* | *sitkensis* |
| *pallidus* | *pensylvanicus* |
| *pennsylvanicus* | *pensylvanicus* |
| *pennsylvannicus* | *pensylvanicus* |
| *pensylvanica* | *pensylvanicus* |
| *pensylvanicus sonorus* | *pensylvanicus* |
| *pleuralis* | *flavifrons* |
| *proximus* | *occidentalis* |
| *prunellae* | *rufocinctus* |
| *putnami* | *kirbiellus* |
| *ridingsii* | *bimaculatus* |
| *rufosuffusus* | *huntii* |
| *scutellaris* | *fraternus* |
| *separatus* | *griseocollis* |
| *sonomae* | *fervidus* |
| *sonorus* | *pensylvanicus* |
| *sonorus flavodorsalis* | *pensylvanicus* |
| *ternarius* var*. expallidus* | *ternarius* |
| *terricola occidentalis* | *occidentalis* |
| *titusi* | *pensylvanicus* |
| *vagans helenae* | *vagans* |
| *vagans sandersoni* | *sandersoni* |
| *vosneskenskii* | *vosnesenskii* |
