# Supplementary material for: Characterizing bumble bee (Bombus) communities in the United States and assessing a conservation monitoring method
Source: Ecol Evol. 2019 Jan 13;9(3):1061–9. doi: 10.1002/ece3.4783 (PMC6374645; doi:10.1002/ece3.4783)
Supplement: Supplementary file 7 [file ECE3-9-1061-s007.docx]

Appendix7_SppOccur. The historic occurrence of each species in each Level III Ecoregion included in this work. Present = a resident species with over 25 specimen records in the assembled data and with records less than 10 years old, Occasional = a species encountered < 25 times in DataSources an ecoregion that may occasionally be observed in an ecoregion, but unlikely to be a resident species, Exotic = records of *Bombus impatiens* in an ecoregion in which it is not native, but has been imported for agricultural pollination and detected outside of containment. Liberal Richness = the number of Present, Occasional, and Exotic species in an ecoregion; Conservative Richness = the number of species designated as Present occurring in an ecoregion. Occurrence determined from records published in DataSources.

| **Species** | **Acadian Plains and Hills** | **Atlantic Coastal Pine Barrens** | **Blue Ridge** | **Cascades** | **Central Basin and Range** | **Chihuahuan Deserts** | **Coast Range** | **Colorado Plateaus** | **Driftless Area** | **Northern Appalachian and Atlantic Maritime Highlands** | **Northern Piedmont** | **Ozark Highlands** | **Piedmont** | **Ridge and Valley** | **Southeastern Plains** | **Southern Coastal Plain** | **Southern Michigan/ Northern Indiana Drift Plains** | **Strait of Georgia/ Puget Lowland** | **Wasatch and Uinta Mountains** | **Willamette Valley** |
| --- | --- | --- | --- | --- | --- | --- | --- | --- | --- | --- | --- | --- | --- | --- | --- | --- | --- | --- | --- | --- |
| ***affinis*** | Present | Present | Present | Absent | Absent | Absent | Absent | Absent | Present | Present | Present | Absent | Present | Present | Present | Absent | Present | Absent | Absent | Absent |
| ***appositus*** | Absent | Absent | Absent | Present | Present | Absent | Absent | Present | Absent | Absent | Absent | Absent | Absent | Absent | Absent | Absent | Absent | Absent | Present | Present |
| ***auricomus*** | Absent | Present | Occasional | Absent | Absent | Absent | Absent | Absent | Present | Absent | Present | Present | Absent | Present | Present | Absent | Occasional | Absent | Absent | Absent |
| ***balteatus*** | Absent | Absent | Absent | Absent | Occasional | Absent | Absent | Occasional | Absent | Absent | Absent | Absent | Absent | Absent | Absent | Absent | Absent | Absent | Absent | Absent |
| ***bifarius*** | Absent | Absent | Absent | Present | Present | Absent | Occasional | Present | Absent | Absent | Absent | Absent | Absent | Absent | Absent | Absent | Absent | Occasional | Present | Absent |
| ***bimaculatus*** | Present | Present | Present | Absent | Absent | Absent | Absent | Absent | Present | Present | Present | Present | Present | Present | Present | Present | Present | Absent | Absent | Absent |
| ***bohemicus*** | Present | Absent | Absent | Absent | Occasional | Absent | Absent | Absent | Absent | Present | Absent | Absent | Absent | Present | Absent | Absent | Present | Absent | Absent | Absent |
| ***borealis*** | Absent | Absent | Absent | Absent | Absent | Absent | Absent | Absent | Absent | Present | Absent | Absent | Absent | Absent | Absent | Absent | Present | Absent | Absent | Absent |
| ***californicus*** | Absent | Absent | Absent | Present | Present | Absent | Present | Present | Absent | Absent | Absent | Absent | Absent | Absent | Absent | Absent | Absent | Present | Present | Present |
| ***caliginosus*** | Absent | Absent | Absent | Present | Absent | Absent | Present | Absent | Absent | Absent | Absent | Absent | Absent | Absent | Absent | Absent | Absent | Absent | Absent | Present |
| ***centralis*** | Absent | Absent | Absent | Occasional | Present | Absent | Absent | Present | Absent | Absent | Absent | Absent | Absent | Absent | Absent | Absent | Absent | Absent | Present | Absent |
| ***citrinus*** | Absent | Present | Present | Absent | Absent | Absent | Absent | Absent | Present | Present | Present | Absent | Present | Present | Present | Absent | Present | Absent | Absent | Absent |
| ***crotchii*** | Absent | Absent | Absent | Absent | Absent | Absent | Absent | Absent | Absent | Absent | Absent | Absent | Absent | Absent | Absent | Absent | Absent | Absent | Absent | Absent |
| ***fervidus*** | Present | Present | Present | Absent | Present | Absent | Present | Present | Occasional | Present | Present | Absent | Absent | Present | Present | Absent | Present | Occasional | Present | Present |
| ***flavidus*** | Absent | Absent | Present | Present | Occasional | Absent | Present | Absent | Absent | Present | Absent | Absent | Occasional | Occasional | Absent | Absent | Occasional | Occasional | Present | Absent |
| ***flavifrons*** | Absent | Absent | Absent | Present | Present | Absent | Present | Occasional | Absent | Absent | Absent | Absent | Absent | Absent | Absent | Absent | Absent | Present | Present | Present |
| ***fraternus*** | Absent | Absent | Absent | Absent | Absent | Absent | Absent | Absent | Absent | Absent | Absent | Present | Occasional | Absent | Present | Present | Absent | Absent | Absent | Absent |
| ***frigidus*** | Absent | Absent | Absent | Absent | Absent | Absent | Absent | Absent | Absent | Present | Absent | Absent | Absent | Absent | Absent | Absent | Absent | Absent | Absent | Absent |
| ***griseocollis*** | Absent | Present | Present | Absent | Present | Absent | Absent | Present | Present | Present | Present | Present | Present | Present | Present | Present | Present | Absent | Present | Present |
| ***huntii*** | Absent | Absent | Absent | Absent | Present | Absent | Absent | Present | Absent | Absent | Absent | Absent | Absent | Absent | Absent | Absent | Absent | Absent | Present | Absent |
| ***impatiens*** | Present | Present | Present | Absent | Absent | Exotic | Absent | Exotic | Present | Present | Present | Present | Present | Present | Present | Present | Present | Absent | Absent | Absent |
| ***insularis*** | Absent | Absent | Absent | Present | Present | Absent | Present | Present | Absent | Absent | Absent | Absent | Absent | Absent | Absent | Absent | Absent | Present | Present | Absent |
| ***melanopygus*** | Absent | Absent | Absent | Present | Present | Absent | Present | Present | Absent | Absent | Absent | Absent | Absent | Absent | Absent | Absent | Absent | Present | Present | Present |
| ***mixtus*** | Absent | Absent | Absent | Present | Present | Absent | Present | Absent | Absent | Absent | Absent | Absent | Absent | Absent | Absent | Absent | Absent | Present | Present | Present |
| ***morrisoni*** | Absent | Absent | Absent | Absent | Present | Absent | Absent | Present | Absent | Absent | Absent | Absent | Absent | Absent | Absent | Absent | Absent | Absent | Present | Absent |
| ***nevadensis*** | Absent | Absent | Absent | Absent | Present | Absent | Absent | Present | Absent | Absent | Absent | Absent | Absent | Absent | Absent | Absent | Absent | Absent | Present | Present |
| ***occidentalis*** | Absent | Absent | Absent | Present | Present | Absent | Present | Present | Absent | Absent | Absent | Absent | Absent | Absent | Absent | Absent | Absent | Present | Present | Present |
| ***pensylvanicus*** | Absent | Occasional | Occasional | Absent | Absent | Present | Absent | Present | Present | Absent | Present | Present | Present | Present | Present | Present | Present | Absent | Absent | Absent |
| ***perplexus*** | Present | Absent | Present | Absent | Absent | Absent | Absent | Absent | Present | Present | Present | Absent | Occasional | Present | Present | Absent | Present | Absent | Absent | Absent |
| ***rufocinctus*** | Occasional | Absent | Absent | Absent | Present | Absent | Absent | Present | Absent | Present | Absent | Absent | Absent | Present | Absent | Absent | Occasional | Absent | Present | Absent |
| ***sandersoni*** | Present | Absent | Present | Absent | Absent | Absent | Absent | Absent | Absent | Present | Absent | Absent | Absent | Present | Absent | Absent | Absent | Absent | Absent | Absent |
| ***sitkensis*** | Absent | Absent | Absent | Present | Absent | Absent | Present | Absent | Absent | Absent | Absent | Absent | Absent | Absent | Absent | Absent | Absent | Absent | Absent | Occasional |
| ***suckleyi*** | Absent | Absent | Absent | Occasional | Occasional | Absent | Absent | Absent | Absent | Absent | Absent | Absent | Absent | Absent | Absent | Absent | Absent | Absent | Present | Absent |
| ***sylvicola*** | Absent | Absent | Absent | Absent | Present | Absent | Absent | Absent | Absent | Absent | Absent | Absent | Absent | Absent | Absent | Absent | Absent | Absent | Present | Absent |
| ***ternarius*** | Present | Absent | Absent | Absent | Absent | Absent | Absent | Absent | Absent | Present | Absent | Absent | Absent | Present | Absent | Absent | Absent | Absent | Absent | Absent |
| ***terricola*** | Present | Absent | Present | Absent | Absent | Absent | Absent | Absent | Absent | Present | Absent | Absent | Occasional | Present | Absent | Absent | Present | Absent | Absent | Absent |
| ***vagans*** | Present | Present | Present | Absent | Absent | Absent | Absent | Absent | Present | Present | Present | Absent | Absent | Present | Absent | Absent | Present | Absent | Absent | Absent |
| ***vandykei*** | Absent | Absent | Absent | Present | Absent | Absent | Present | Absent | Absent | Absent | Absent | Absent | Absent | Absent | Absent | Absent | Absent | Absent | Absent | Absent |
| ***variabilis*** | Absent | Absent | Absent | Absent | Absent | Absent | Absent | Absent | Absent | Absent | Absent | Present | Absent | Absent | Absent | Present | Absent | Absent | Absent | Absent |
| ***vosnesenskii*** | Absent | Absent | Absent | Present | Absent | Absent | Present | Absent | Absent | Absent | Absent | Absent | Absent | Absent | Absent | Absent | Absent | Present | Absent | Present |
| **Liberal Richness*** | **11** | **9** | **13** | **15** | **20** | **2** | **13** | **17** | **10** | **16** | **10** | **7** | **10** | **16** | **10** | **6** | **15** | **10** | **18** | **12** |
| **Conservative Richness** | **10** | **8** | **11** | **13** | **16** | **1** | **12** | **14** | **9** | **16** | **10** | **7** | **6** | **15** | **10** | **6** | **12** | **7** | **18** | **11** |
|  |  |  |  |  |  |  |  |  |  |  |  |  |  |  |  |  |  |  |  |  |
| ***Includes Exotic and Occassional species** |  |  |  |  |  |  |  |  |  |  |  |  |  |  |  |  |  |  |  |  |
