# Supplementary material for: Characterizing bumble bee (Bombus) communities in the United States and assessing a conservation monitoring method
Source: Ecol Evol. 2019 Jan 13;9(3):1061–9. doi: 10.1002/ece3.4783 (PMC6374645; doi:10.1002/ece3.4783)

Appendix8_NMDS. Output from NMDS analysis in two graphics. Appendix 8a is an NMDS showing the species composition of sites by the species detected at those sites. Appendix 8b is an NMDS of the data grouped by ecoregion to compare the species composition detected at that level.


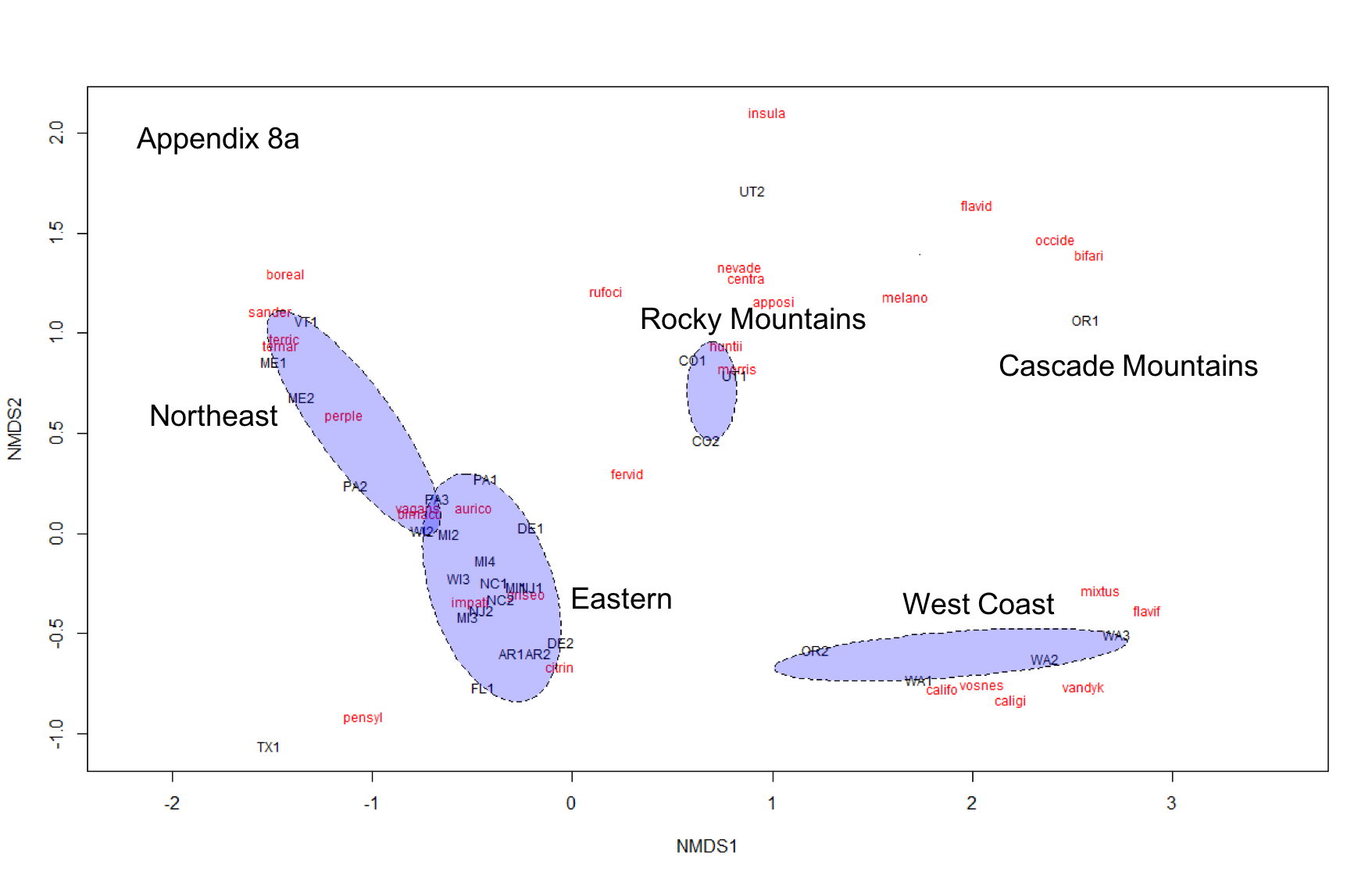


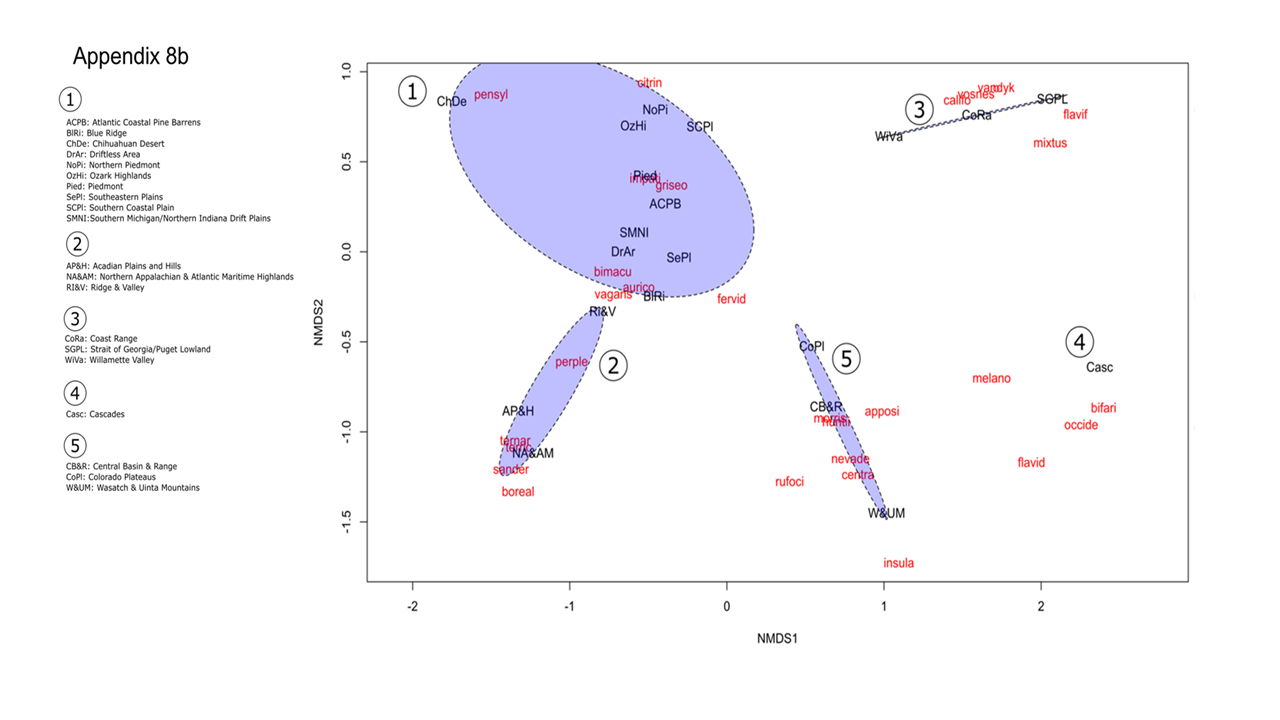

Supplement: Supplementary file 8 [file ECE3-9-1061-s008.docx]
